# *Lactiplantibacillus plantarum* Strain 06CC2 Attenuates Fat Accumulation and Modulates the Gut Microbiota in a Mouse Model of Early-Stage Diet-Induced Obesity

**DOI:** 10.3390/nu17243855

**Published:** 2025-12-10

**Authors:** Tatsuya Matsusaki, Chisato Takakura, Kaho Ichitani, Chuluunbat Tsend-Ayush, Hiroaki Kataoka, Tsuyoshi Fukushima, Junko Kurogi, Kazuo Nishiyama, Kenjirou Ogawa, Takuo Shinyama, Tomoki Nakano, Masao Yamasaki

**Affiliations:** 1 Graduate School of Agriculture, University of Miyazaki, 1-1 Gakuen Kibanadai-nishi, Miyazaki 889-2192, Japan; t-matsusaki@dairy-milk.co.jp (T.M.); gc21033@student.miyazaki-u.ac.jp (C.T.); jm1811.k@outlook.jp (K.I.); nishiyam@cc.miyazaki-u.ac.jp (K.N.); ogawa.kenjirou.u2@cc.miyazaki-u.ac.jp (K.O.); 2 Research and Development Division, Minami Nihon Rakuno Kyodo Co., Ltd., 5282 Takagi, Miyakonojo 885-0003, Japan; t-shinyama@dairy-milk.co.jp (T.S.); t-nakano@dairy-milk.co.jp (T.N.); 3 School of Food, Light Industry and Design, Mongolian University of Science and Technology, Baga Toiruu, Sukhbaatar District, P.O. Box 46/520, Ulaanbaatar 14191, Mongolia; tsend@must.edu.mn; 4 Section of Oncopathology and Morphological Pathology, Department of Pathology, Faculty of Medicine, University of Miyazaki, 5200 Kihara, Kiyotake, Miyazaki 889-1692, Japan; mejina@med.miyazaki-u.ac.jp (H.K.); tsufuku@icloud.com (T.F.); junko_kurogi@med.miyazaki-u.ac.jp (J.K.)

**Keywords:** *Lactiplantibacillus plantarum*, probiotics, diet-induced obesity, mice, gut microbiota, 16S rRNA sequencing, adipose tissue inflammation, hepatic steatosis

## Abstract

**Background/Objectives**: The increase in the global prevalence of obesity has created a need for safe and effective preventive strategies. Probiotics have gained attention for their potential to modulate the gut microbiota and improve metabolic health. In this study, we examined the anti-obesity effects of *Lactiplantibacillus plantarum* strain 06CC2 (LP06CC2) in a mouse model of mild diet-induced obesity that mimics early-stage metabolic imbalance without significant body weight gain. **Methods**: Mice were fed a high-fat diet for 8 weeks, with or without LP06CC2 supplementation. Biochemical assays were used to determine the metabolic effects of LP06CC2, and 16S rRNA sequencing was performed to analyze the gut microbiota. **Results**: LP06CC2 attenuated epididymal fat accumulation and adipocyte hypertrophy, improved the gene expression profiles related to lipid metabolism and inflammation in adipose tissue, and reduced early hepatic steatosis. 16S rRNA sequencing revealed that LP06CC2 modulated the diversity and composition of the gut microbiota, notably suppressing HFD-induced increases in *Mucispirillum schaedleri* and other taxa associated with inflammation. LP06CC2-treated mice exhibited higher alpha diversity and partial restoration of their microbial profiles toward those of the normal diet-fed animals. LP06CC2 also downregulated pro-inflammatory cytokines and genes related to lipid uptake while modulating markers of thermogenesis and lipolysis. **Conclusions**: These findings indicate that LP06CC2 can prevent fat accumulation and gut dysbiosis in the pre-obese state, supporting its potential as a functional food ingredient for early intervention in obesity. Further human trials and studies using advanced obesity models are warranted to confirm its efficacy and elucidate its underlying mechanisms of action.

## 1. Introduction

With the global incidence of obesity steadily increasing, the need for safe and effective therapeutic strategies has become a pressing public health issue. The World Health Organization reports that worldwide obesity has nearly tripled since 1975, with over 1 billion people, including 650 million adults, 340 million adolescents, and 39 million children, classified as obese as of 2022 [1]. This epidemic is closely associated with the development of metabolic disorders, including type 2 diabetes, cardiovascular disease, and non-alcoholic fatty liver disease (NAFLD), imposing substantial economic and social burdens on healthcare systems worldwide [2,3].

Probiotics, defined as live microbes that promote health when taken in adequate doses, have been widely studied for their therapeutic potential [4]. Probiotic-mediated modulation of the gut microbiota has attracted growing attention as a potential intervention for improving metabolic health without adversely affecting quality of life [5,6]. The human gut microbiota, comprising approximately 40 trillion microorganisms, plays a crucial role in host metabolism through various mechanisms, including energy harvest from dietary components, regulation of intestinal permeability, and modulation of inflammatory responses [7,8]. Dysbiosis, characterized by an imbalance in gut microbial composition, has been implicated in the pathogenesis of obesity and related metabolic disorders [9,10,11].

Lactic acid bacteria (LAB), particularly those belonging to the *Lactobacillus* genus, represent one of the most extensively studied probiotic groups for metabolic health applications. These Gram-positive, facultatively anaerobic bacteria exert their beneficial effects through multiple mechanisms. Among various probiotic strains, *Lactiplantibacillus plantarum* has shown anti-obesity effects in several animal models through its unique metabolic capabilities and host-microbe interactions. *L. plantarum*, an extensively studied probiotic microorganism, confers a spectrum of beneficial effects on host health, with a notable capacity to counteract the onset and progression of various chronic metabolic diseases [12]. This species exhibits remarkable genetic diversity, with pan-genome analyses revealing extensive strain-specific gene repertoires that confer unique metabolic capabilities [13]. Its robust nature allows survival through harsh gastrointestinal conditions, including low pH environments and bile salt exposure, enabling effective colonization and temporary establishment in the human gut [14]. The species demonstrates remarkable metabolic flexibility, capable of utilizing diverse carbon sources and producing various bioactive metabolites, including short-chain fatty acids (SCFAs), bacteriocins, and organic acids, which contribute to its probiotic efficacy [15].

The *L. plantarum* strain 06CC2 (LP06CC2) used in this study was originally isolated from traditional Mongolian dairy products [16]. Previous studies have demonstrated its diverse probiotic properties, including its suppression of inflammation in a mouse model of ulcerative colitis [17] and reduction in hepatic cholesterol levels in mice fed a high-cholesterol diet [18]. Recently, LP06CC2 was shown to alleviate hyperuricemia symptoms in a potassium oxonate-induced high-purine mouse model [19]. Moreover, we previously showed that LP06CC2 exerts anti-obesity effects in BALB/c mice with diet-induced obesity [20], suggesting strain-specific metabolic benefits.

In the present study, we evaluated the effects of an 8-week treatment of LP06CC2 on obesity-related parameters in C57BL/6J mice, a well-established model for HFD-induced obesity research, to elucidate the molecular mechanisms underlying its prevention of early-stage obesity, focusing on gut microbiota dynamics. Our findings highlight the potential use of this probiotic strain as part of a lifestyle-related disease prevention strategy.

## 2. Materials and Methods

### 2.1. Preparation of LP06CC2

LP06CC2, which was isolated from traditional Mongolian dairy products, is described as a probiotic owing to its resistance to bile and gastric acids and its ability to adhere to Caco-2 cells [16]. LP06CC2 cells were pre-cultured at 37 °C for 18 h in de Man–Rogosa–Sharpe (MRS) broth (Merck Millipore, Darmstadt, Germany). Then, 20 mL of the pre-culture suspension was transferred to 2 L of fresh MRS broth and incubated at 37 °C for 18 h. After fermentation, bacterial growth was confirmed by measuring the optical density at 660 nm (OD_660_ = ~5.0–5.5). Subsequently, the bacterial cells were centrifuged at 1500× *g* for 5 min, washed twice with phosphate-buffered saline, and lyophilized, and the resultant powder was stored at −80 °C until use.

### 2.2. Experimental Animals and Dietary Intervention Protocol

The animal experiments were approved by the ethics committee of the University of Miyazaki (Approval No. 2017-006-6, Approval Date: 14 June 2021) and conducted in accordance with Chapters 4 (Conduct of Animal Experiments, etc.), 5 (Facilities, etc.), 6 (Breeding and Storage of Laboratory Animals), 8 (Safety Management), and 9 (Education and Training) of the University of Miyazaki Animal Experiment Regulations. Five-week-old male C57BL/6J mice weighing 18–23 g were obtained from the Japan SLC Corporation (Hamamatsu, Japan). After 7 days of pre-breeding and from 6 weeks of age, the mice were divided into the following four groups: normal diet (ND), normal diet plus LP06CC2 (ND + LP), high-fat diet (HFD), and high-fat diet plus LP06CC2 (HFD + LP). The mice had free access to their respective diets for 8 weeks. The ND was a standard diet based on AIN-93G, whereas the HFD was a variation in the standard diet mixed with lard at 330 g/kg (Table 1). LP06CC2 was mixed into the feed at a concentration of 0.1% (*w*/*w*), as described in our previous study [20]. The diets were preserved at −80 °C. The mice were fed and provided water ad libitum. The fecal samples were collected on days 53–56, then individually pooled and stored at −30 °C. The body weights of the mice were measured once weekly over the 8-week feeding period. To assess statistical differences between the changes in body weight, the area under the curve (AUC) was used. The food intake was determined by measuring the remaining feed every two days for each mouse and calculating the difference from the amount provided.

### 2.3. Feeding Termination and Biological Sample Collection

At the end of the 8-week feeding period, the mice were fasted for 15 h, anesthetized with an intraperitoneal injection of medetomidine (0.75 mg/kg), midazolam (4.0 mg/kg), and butorphanol (5.0 mg/kg). Blood was collected via cardiac puncture and centrifuged at 100× *g* for 20 min at 4 °C. The supernatant was collected as plasma and stored at −80 °C until further analysis. The liver, small intestine, cecum, large intestine, epididymal fat, and perirenal fat were excised and weighed. The livers were fixed in 10% neutral-buffered formalin. The epididymal fat was divided as follows: one portion was preserved in RNAlater reagent (Qiagen, Hilden, Germany) and stored at −80 °C for subsequent RNA extraction, whereas the remainder was fixed in formalin for histological analysis.

### 2.4. Biochemical Analysis of Plasma and Liver Samples

The plasma and hepatic levels of triglycerides (TGs) and total cholesterol (TC) as well as plasma levels of aspartate aminotransferase (AST), alanine aminotransferase (ALT), and fibroblast growth factor 21 (FGF21) were measured using commercial kits: TG E-test, Cholesterol E-test, and Transaminase CII-test (Wako Pure Chemical Industries, Osaka, Japan), and FGF-21 ELISA Kit (Proteintech Group, Inc., Tokyo, Japan), respectively. The liver tissue samples (100 mg) were homogenized at 4000 rpm for 3 min at 4 °C using a Micro Smash MS-100R bead cell disruptor (TOMY SEIKO Co., Ltd., Tokyo, Japan). Lipids were extracted using chloroform–methanol (2:1, *v*/*v*), evaporated under a stream of nitrogen gas, and resuspended in 2-propanol containing 5% Tween-20 (*v*/*v*) for subsequent TG and TC quantification. The levels of high-density lipoprotein-cholesterol (HDL-C), low-density lipoprotein-cholesterol (LDL-C), non-esterified fatty acid (NEFA), uric acid (UA), and total bile acid (TBA) in the plasma were measured by Oriental Yeast Co., Ltd. (Shiga, Japan).

### 2.5. Histological Evaluation of Adipose and Liver Tissues

Epididymal fat and liver tissue samples were first embedded in paraffin. Subsequently, the tissue blocks were cut into 5 μm sections, and these were then stained with hematoxylin and eosin (H&E). The epididymal fat area was quantified by measuring a minimum of 100 cells per tissue sample from randomly selected microscopic fields. The data were analyzed and expressed as frequency histograms using cellSens software (Standard 1.15, Olympus, Tokyo, Japan). However, accurate automated quantification of hepatic lipid droplets using available segmentation tools was not possible, as the tools misidentified non–lipid areas, including vascular lumina such as hepatic arteries, portal veins, and sinusoids, as lipid vacuoles. Therefore, using H&E-stained liver sections, histological evaluation of hepatic steatosis was performed according to the Nonalcoholic Fatty Liver Disease (NAFLD) Activity Score (NAS) system [21]. The NAS was calculated as the sum of the scores for steatosis (0–3), lobular inflammation (0–3), and hepatocellular ballooning (0–2), following the criteria established by the Nonalcoholic Steatohepatitis Clinical Research Network. The semi-quantification scoring was conducted in a blinded manner by professional pathologists.

### 2.6. Reverse Transcription Quantitative Real-Time Polymerase Chain Reaction

Total RNA was extracted from the epididymal fat using the RNeasy Lipid Tissue Mini Kit (Qiagen, Hilden, Germany) according to the manufacturer’s instructions. The concentration and purity of the RNA were measured using a NanoDrop 2000 spectrophotometer (Thermo Fisher Scientific, Waltham, MA, USA). Equal amounts of the extracted RNA (2 μg) were reverse-transcribed using the ReverTra Ace α Kit (Toyobo Co., Ltd., Osaka, Japan) according to the manufacturer’s instructions. Quantitative PCR was performed using the PowerUp SYBR Green Master Mix (Thermo Fisher Scientific) on a 7300 Real-Time PCR system (Applied Biosystems, Tokyo, Japan). The reaction was performed for an initial denaturation at 95 °C for 10 min, followed by 40 PCR amplification cycles of 95 °C for 5 s, 58 °C for 30 s, and 72 °C for 30 s. The dissociation stage was analyzed at 95 °C for 15 s, followed by 1 cycle of 60 °C for 1 min and 95 °C for 15 s. The primers used are listed in Supplementary Table S1. *Gapdh* was selected as the reference gene after preliminary validation confirmed stable expression across experimental groups. The gene expression levels were normalized to that of *Gapdh* and analyzed using the 2^–ΔΔCt^ method.

### 2.7. Microbiota Analysis via 16S Ribosomal Ribonucleic Acid Sequencing

Total DNA was extracted from the fecal samples, and the V3–V4 region of the 16S rRNA gene was amplified and sequenced using the Illumina MiSeq platform (2 × 300 bp, paired-end reads). The sequencing was performed by Techno Suruga Laboratory Co., Ltd. (Shizuoka, Japan) [22]. The raw FASTQ files were provided by the vendor and processed in-house using QIIME 2 (version 2023.2) [23]. After quality filtering, denoising, and chimera removal using DADA2 [24], feature tables were generated. Taxonomic assignment was performed using the Genome Taxonomy Database-based Species-Level Reference (GSR-DB) [25]. Alpha diversity metrics (i.e., observed features, Chao1 richness estimator [26], and Shannon diversity index [27]) were calculated using QIIME 2. Beta diversity was assessed on the basis of the weighted UniFrac [28], Bray–Curtis [29], and Jaccard [30] distances and visualized using principal coordinate analysis (PCoA). Statistical comparisons among groups were performed using permutational multivariate analysis of variance (PERMANOVA) with 999 permutations [31]. The relative abundance of *L. plantarum* was also evaluated, and key taxa (e.g., *Mucispirillum schaedleri*, *Adlercreutzia mucosicola*, Lachnospiraceae, and Oscillospiraceae) showing group-dependent shifts were extracted for further analysis.

### 2.8. Statistical Analysis

Data are expressed as the mean ± standard error of the mean. All statistical analyses were performed using GraphPad Prism 10.2.1 (GraphPad Software, La Jolla, CA, USA). One-way analysis of variance followed by Tukey’s multiple comparison test was used for comparing more than two groups. Differences with a *p*-value of less than 0.05 were considered statistically significant. Spearman’s rank correlation analysis was performed to examine associations between epididymal fat parameters (mass, adipocyte area, and mRNA expression of lipid metabolism genes) and the relative abundance of key fecal microbial taxa. Correlation coefficients (ρ) and corresponding *p*-values were calculated, with *p* < 0.05 considered statistically significant.

## 3. Results

### 3.1. Physiological Parameters and Tissue Masses

The physiological parameters and food intake data are summarized in Table 2. No significant differences in final body weight were found among the groups. Although the food and calorie intakes in the HFD and HFD + LP groups were lower than those in the ND and ND + LP groups, LP06CC2 did not influence food intake. The changes in body weight over time are shown in Supplementary Figure S1A. Regarding the AUC of changes in body weight, no significant differences were observed among the groups (Supplementary Figure S1B). The tissue masses are listed in Table 3. In the HFD group, the epididymal fat weight was significantly higher than that in the ND and ND + LP groups. However, this significant difference was not observed between the HFD + LP and the ND or ND + LP groups. Although perirenal adipose tissue exhibited a weight trend similar to that of epididymal fat, the difference was not statistically significant. Moreover, the masses of the liver, small intestine, cecum, and large intestine did not differ significantly among the groups.

### 3.2. Plasma and Hepatic Lipid Profiles

The levels of various biochemical markers in the plasma and liver samples are summarized in Table 4. The plasma LDL-C levels were significantly higher in the HFD group than in the ND + LP group. Additionally, the LDL-C levels tended to be lower in the ND + LP group than in the ND group as well as lower in the HFD + LP group than in the HFD group. Moreover, the plasma and hepatic TG levels tended to be lower in the HFD + LP group than in the HFD group, but the differences were not statistically significant. Similarly, plasma NEFA levels were significantly increased in the HFD group compared to the ND group; however, LP supplementation did not significantly alter NEFA levels. Plasma FGF21 levels were significantly elevated in the HFD and ND + LP groups compared to the ND group. While the HFD + LP group exhibited the highest FGF21 concentration, no significant difference was observed from the HFD group.

### 3.3. Histopathological Changes in the Epididymal Fat and Liver

**Figure 1:**
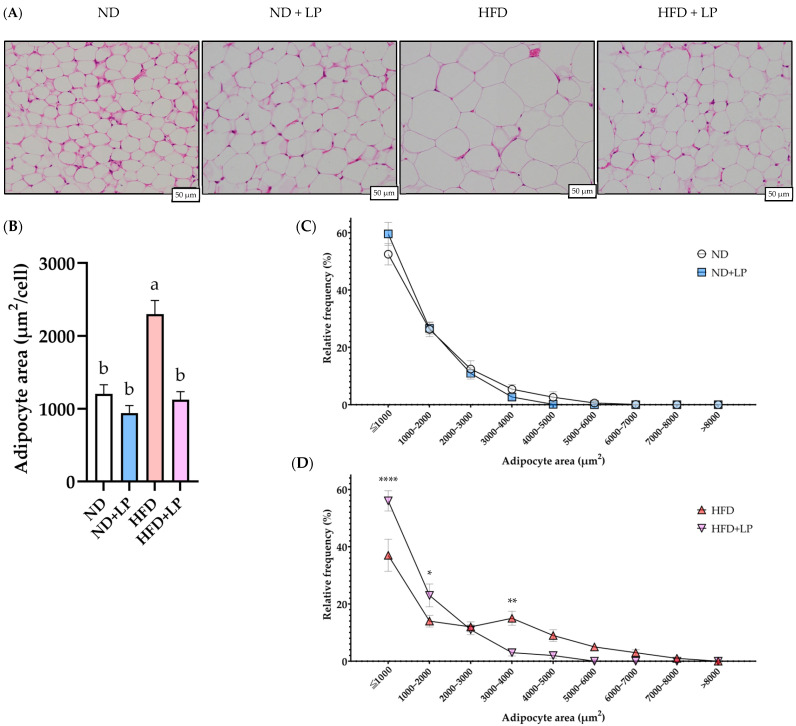
Effect of LP06CC2 on epididymal fat morphology. (**A**) H&E staining pictures of representative epididymal fat tissues of different groups (scale bar = 50 µm, magnification 200×), (**B**) average adipocyte area, and (**C**,**D**) histogram of adipocyte area distribution. Data are presented as the mean ± SEM for eight mice per group. ^a, b^ Values without any common letters are significantly different from each other (*p* < 0.05). * *p* < 0.05, ** *p* < 0.01, and **** *p* < 0.0001. ND—normal diet; HFD—high-fat diet; LP—*Lactiplantibacillus plantarum* strain 06CC2; H&E—hematoxylin and eosin.

**Figure 2:**
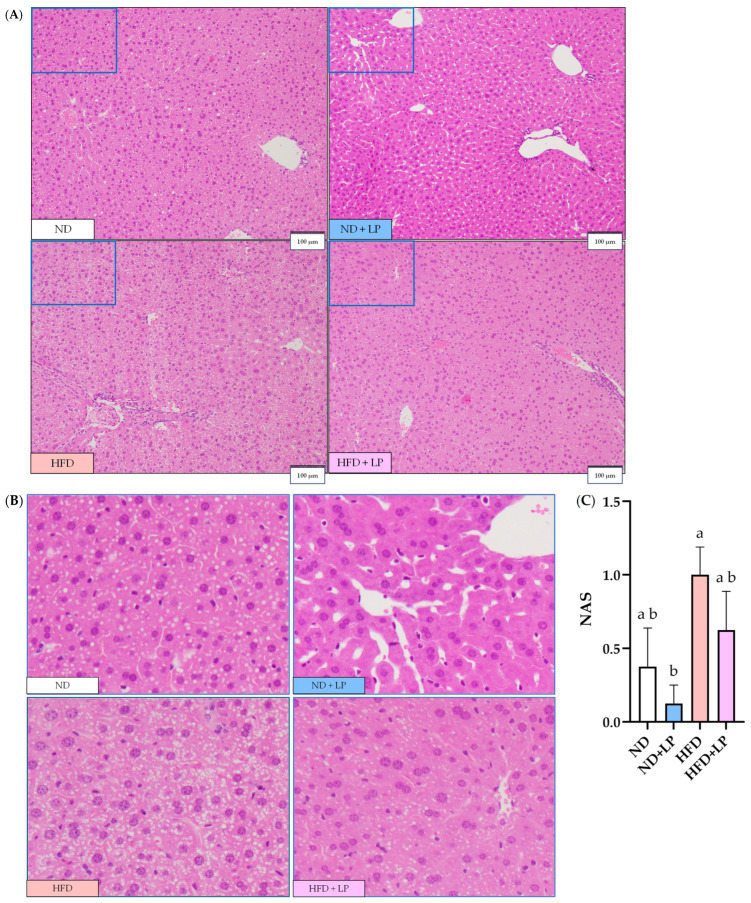
Effect of LP06CC2 on liver morphology. (**A**) H&E staining pictures of representative liver tissues of different groups (scale bar = 100 µm, magnification 100 ×). (**B**) Higher magnification of the boxed areas in (**A**). (**C**) NAS. Data are presented as the mean ± SEM for eight mice per group. ^a, b^ Values without any common letters are significantly different from each other (*p* < 0.05). ND—normal diet; HFD—high-fat diet; LP—*Lactiplantibacillus plantarum* strain 06CC2; H&E—hematoxylin and eosin; NAS—non-alcoholic fatty liver disease (NAFLD) activity score.

H&E staining of the epididymal fat samples showed enlarged adipocytes in the HFD group compared with those in the ND and ND + LP groups (Figure 1A,B). By contrast, adipocytes in the HFD + LP group were smaller and showed a shift toward a reduction in cell size distribution (Figure 1C). In the H&E-stained liver sections, numerous small lipid droplets were observed in the HFD group, but these were not observed in the ND and ND + LP groups (Figure 2A). The histological observation revealed a visual tendency toward fewer small lipid droplets in the HFD + LP group compared with the HFD group. The NAS was significantly higher in the HFD group than in the ND + LP group, whereas it tended to be lower in the HFD + LP group than in the HFD group (Figure 2B).

### 3.4. Modulation of Lipid Metabolism-Related Gene Expression in Epididymal Fat by Diet and LP06CC2 Treatment

**Figure 3:**
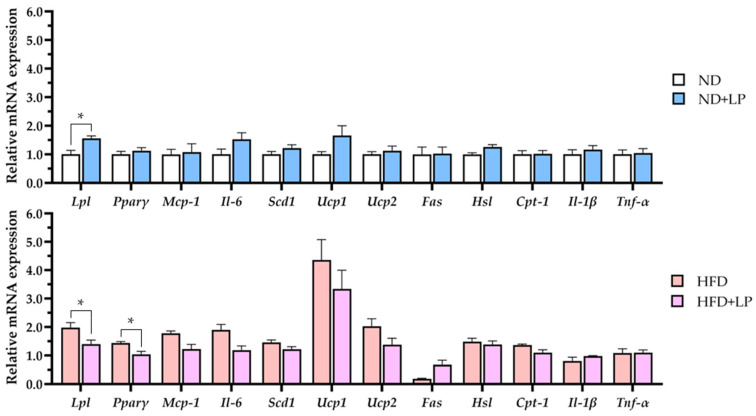
Effect of LP06CC2 on mRNA expression of lipid metabolism genes. Data are presented as the mean ± SEM for eight mice per group. * *p* < 0.05. ND—normal diet; HFD—high-fat diet; LP—*Lactiplantibacillus plantarum* strain 06CC2; *Lpl*—lipoprotein lipase; *Pparγ*—peroxisome proliferator-activated receptor gamma; *Mcp-1*—monocyte chemoattractant protein-1; *Il-6*—interleukin-6; *Scd1*—stearoyl-CoA desaturase; *Ucp1* and *Ucp2*—uncoupling proteins 1 and 2; *Fas*—fatty acid synthase; *Hsl*—hormone-sensitive lipase; *Cpt-1*—carnitine palmitoyltransferase 1.

The expression of lipid metabolism-related genes in the epididymal fat was modulated by dietary conditions and LP06CC2 treatment (Figure 3). Compared with those in the ND group, the expression levels of *Lpl* (encoding lipoprotein lipase) and *Pparγ* (encoding peroxisome proliferator-activated receptor gamma) were significantly increased in the HFD group and significantly decreased in the HFD + LP group.

The expression levels of *Mcp-1*, *Il-6*, *Scd1*, *Ucp1*, and *Ucp2* (encoding monocyte chemoattractant protein-1, interleukin-6, stearoyl-CoA desaturase 1, and uncoupling proteins 1 and 2, respectively) were significantly elevated in the HFD group, whereas these increases were negated in the HFD + LP group. The expression levels of *Fas* (encoding fatty acid synthase) were significantly lower in the HFD group than in the ND group, but this reduction was negated by LP06CC2 treatment. Additionally, the expression levels of *Hsl* (encoding hormone-sensitive lipase) were significantly increased in the HFD group and decreased in the HFD + LP group. Similarly, the expression of levels *Cpt-1 (encoding carnitine palmitoyltransferase-1)* was increased in the HFD group but decreased following LP06CC2 treatment. The expression levels of *Il-1β* and *Tnf-α* (encoding tumor necrosis factor-alpha) were not significantly different among groups.

### 3.5. Changes in Fecal Microbiota in Response to Diet and LP06CC2 Treatment

**Figure 4:**
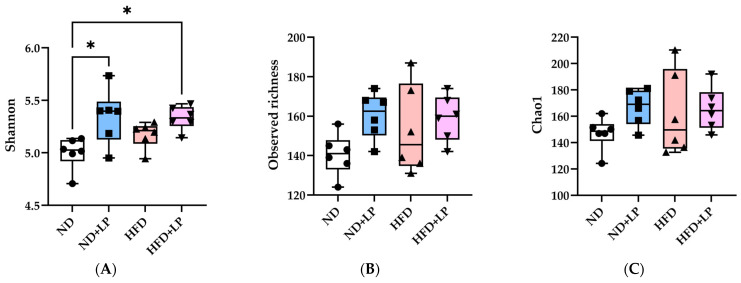
Effect of LP06CC2 on fecal microbial alpha diversity. (**A**) Shannon index, (**B**) observed richness, and (**C**) Chao1 richness estimator. Data are presented as box plots showing median, quartiles, and outliers for six mice per group. * *p* < 0.05. ND—normal diet; HFD—high-fat diet; LP—*Lactiplantibacillus plantarum* strain 06CC2.

The effects of diet and LP06CC2 on the fecal microbiota were analyzed via 16S rRNA amplicon sequencing. The alpha diversity of the gut microbiota was assessed on the basis of the Shannon diversity index, observed richness, and the Chao1 richness estimator (Figure 4). The Shannon index was significantly increased in both the ND + LP and HFD + LP groups compared with that in the ND group, indicating that LP06CC2 treatment enhanced the microbial diversity. By contrast, no significant differences in observed richness or Chao1 richness estimator values were observed among the groups, suggesting that richness was not markedly affected.

**Figure 5:**
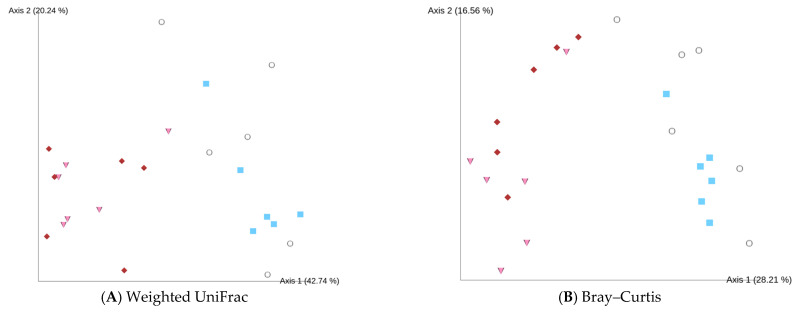

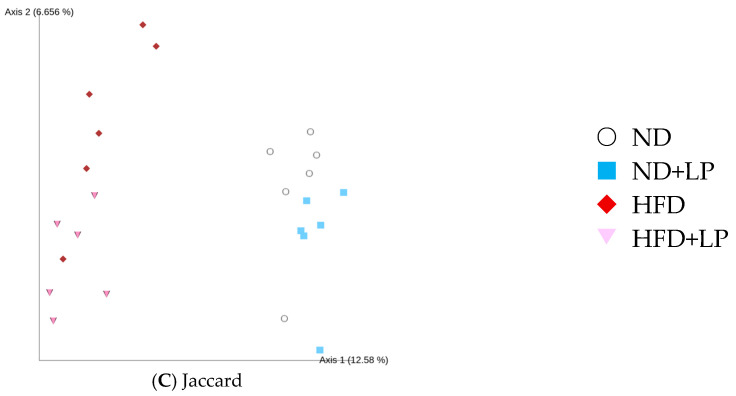
Effect of LP06CC2 on fecal microbial beta diversity. Principal coordinate analysis plots based on (**A**) weighted UniFrac, (**B**) Bray–Curtis, and (**C**) Jaccard distances for six mice per group. ND—normal diet; HFD—high-fat diet; LP—*Lactiplantibacillus plantarum* strain 06CC2.

With regard to the beta diversity, no significant differences in the weighted UniFrac distances were observed following LP supplementation, implying a conserved phylogenetic structure among the groups (Figure 5A). By contrast, Bray–Curtis and Jaccard distance-based PCoAs revealed significant group separation (PERMANOVA, *p* < 0.05), indicating that the microbial community composition had changed in terms of relative abundance and the presence/absence of specific taxa (Figure 5B,C).

**Figure 6:**
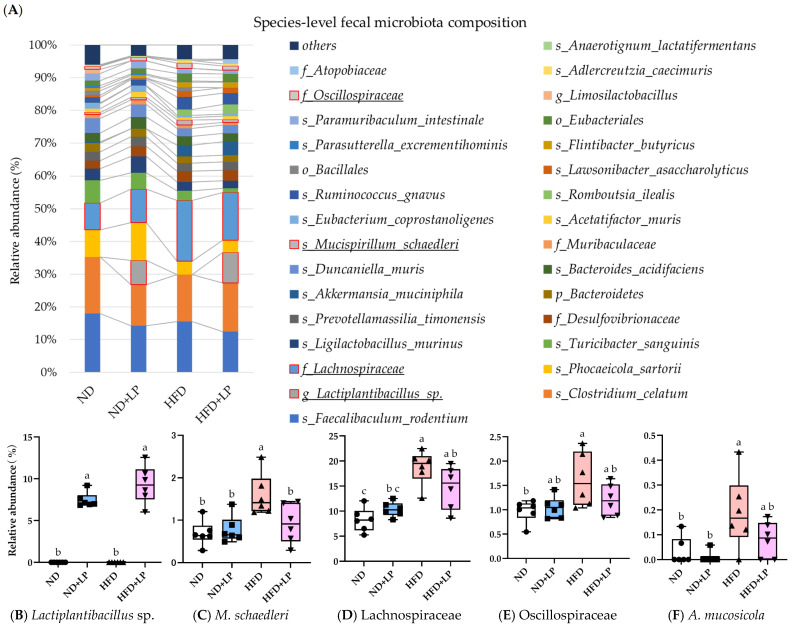
Effect of LP06CC2 on species-level fecal microbiota abundance. (**A**) Relative abundance of the top 30 species (remaining species grouped as ‘others’). (**B**–**F**) Individual taxa abundance: (**B**) *Lactiplantibacillus* sp., (**C**) *Mucispirillum schaedleri*, (**D**) Lachnospiraceae, (**E**) Oscillospiraceae, and (**F**) *Adlercreutzia mucosicola*. Data in panels (**B**–**F**) are presented as box plots showing median, quartiles, and outliers for 6 mice per group. ^a, b, c^ Values without any common letters are significantly different from each other (*p* < 0.05). ND—normal diet; HFD—high-fat diet; LP—*Lactiplantibacillus plantarum* strain 06CC2.

The results of the species-level fecal microbiota composition are shown in Figure 6A and Supplementary Table S2. Notably, *L. plantarum* was detected exclusively in the LP-treated groups (ND + LP and HFD + LP), confirming the effective delivery of the bacterium via dietary supplementation with 0.1% LP06CC2 (Figure 6B). This strain was absent in the ND and HFD groups, which is consistent with previous findings that *L. plantarum* is not a natural resident of the murine gut under specific pathogen-free (SPF) conditions. Among the taxa modulated by the HFD and LP, *M. schaedleri* was markedly increased in the HFD group relative to its abundance in the other three groups (Figure 6C). LP06CC2 treatment suppressed this increase, restoring the species to a level comparable to that in the ND group. Similar patterns were observed for members of the Lachnospiraceae and Oscillospiraceae families as well as *A. mucosicola* (Figure 6D–F). These bacteria, which have previously been implicated in mucosal inflammation and metabolic dysregulation, were enriched by the HFD and normalized by the LP06CC2 treatment.

### 3.6. Correlation Between Epididymal Fat Parameters and Fecal Microbiota According to Diet and LP06CC2 Treatment

**Figure 7:**
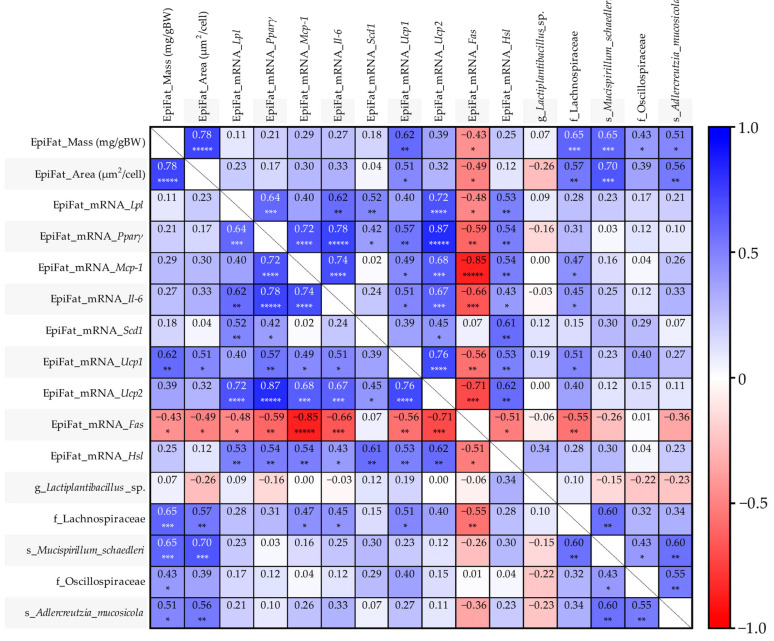
Spearman’s rank correlation between the epididymal fat parameters (mass, adipocyte area, and lipid metabolism genes) and species-level fecal microbiota relative abundance (g_*Lactiplantibacillus* sp., f_Lachnospiraceae, s_*Mucispirillum schaedleri*, f_Oscillospiraceae, and s_*Adlercreutzia mucosicola*) in HFD-induced mice. Values represent Spearman’s coefficients (ρ), with asterisks indicating significance levels: * *p* < 0.05, ** *p* < 0.01, *** *p* < 0.001, **** *p* < 0.0001, and ***** *p* < 0.00001. Epi, epididymal; BW—body weights; *Lpl*—lipoprotein lipase; *Pparγ*—peroxisome proliferator-activated receptor gamma; *Mcp-1*—monocyte chemoattractant protein-1; *Il-6*—interleukin-6; *Scd1*—stearoyl-CoA desaturase; *Ucp1* and *Ucp2*—uncoupling proteins 1 and 2; *Fas*—fatty acid synthase; *Hsl*—hormone-sensitive lipase.

Spearman’s correlation analysis examined relationships between epididymal fat parameters and predominant microbial taxa (Figure 7). Fat mass and adipocyte area showed a positive correlation (ρ = 0.78, *p* < 0.00001). Among lipid metabolism genes, *Pparγ* correlated strongly with *Ucp2* (ρ = 0.87, *p* < 0.00001), *Il-6* (ρ = 0.78, *p* < 0.00001), *Mcp-1* (ρ = 0.72, *p* < 0.0001), and *Lpl* (ρ = 0.64, *p* < 0.001). The inflammatory marker *Mcp-1* was positively correlated with *Il-6* (ρ = 0.74, *p* < 0.0001), and *Ucp2* (ρ = 0.68, *p* < 0.001), but exhibited a very strong negative correlation with the lipogenic gene *Fas* (ρ = −0.85, *p* < 0.00001). *Il-6* also showed a positive association with *Ucp2* (ρ = 0.67, *p* < 0.001) and a negative association with *Fas* (ρ = −0.66, *p* < 0.001). Furthermore, *Ucp1* and *Ucp2* showed a strong positive association (ρ = 0.76, *p* < 0.0001), while *Ucp2* was inversely correlated with *Fas* (ρ = −0.71, *p* < 0.001). Two taxa exhibited robust positive associations with adiposity: Lachnospiraceae (fat mass: ρ = 0.65, *p* < 0.001) and *M. schaedleri* (fat mass: ρ = 0.65, *p* < 0.001; fat area: ρ = 0.70, *p* < 0.001).

## 4. Discussion

The viscosity of the HFD used in this study was relatively high, which has been reported to slightly reduce food intake compared with a normal standard diet [20]. Nevertheless, previous studies have demonstrated that this HFD leads to significant fat accumulation without affecting the body weight [32,33]. Such fat accumulation in the absence of significant weight gain reflects a state of mild obesity. Therefore, in this study, we investigated whether the preventive strategy of LP06CC2 administration could influence physiological outcomes in this mild obesity model. Our results showed that the HFD-fed mice tended to have increased epididymal fat tissue masses and plasma LDL-C levels compared with their ND-fed counterparts, whereas these increases tended to be negated in the mice fed both the HFD and LP. In contrast, plasma and hepatic TGs levels were lower than expected, differing from our previous study [20]. These contrasting results could be explained by metabolic variations between different mouse strains [34,35]. These findings indicate that our model effectively replicates a mild obesity state and further suggest that it may be useful for evaluating the preventive potential of functional food components, such as probiotics. The viable counts of LP06CC2 in the feed were approximately 6.8 × 10^7^ colony-forming units (CFU)/g in the ND + LP group and 6.4 × 10^7^ CFU/g in the HFD + LP group. This concentration is lower than the common doses of 10^8^–10^9^ CFU/g typically employed in many other studies utilizing *Lactobacillus* strains [33,36,37,38,39,40]. Therefore, the observed efficacy of LP06CC2 at this relatively low concentration is significant and underscores its potency.

Interestingly, plasma FGF21 concentrations tended to increase in all experimental groups (ND + LP, HFD, and HFD + LP) compared with the ND group. FGF21 is an endocrine hormone that improves glucose and lipid metabolism and has been proposed as a potential therapeutic target for obesity-related metabolic disorders. Previous studies have shown that circulating FGF21 levels are elevated in obese mice fed a high-fat or high-carbohydrate diet, suggesting the development of FGF21 resistance during the onset of obesity [41]. Therefore, the increase in plasma FGF21 observed in the HFD group may reflect a compensatory response to FGF21 resistance induced by chronic overnutrition. In contrast, the further elevation of FGF21 in LP-treated mice might indicate partial restoration of FGF21 signaling or improved metabolic adaptation mediated by LP. A recent study using db/db mice demonstrated that *Lactobacillus johnsonii* upregulated hepatic FGF21 expression, thereby improving lipid and glucose metabolism [42]. While the experimental models differ, our findings are consistent in suggesting that probiotics may modulate hepatic FGF21 expression. Thus, the observed rise in FGF21 in LP-treated mice could reflect an adaptive hormonal response contributing to the amelioration of dyslipidemia rather than a simple compensatory elevation. Additionally, plasma TBA levels tended to be higher in the HFD group but slightly decreased in the HFD + LP group. Given that *L. plantarum* strains are known to modulate bile acid metabolism through bile salt hydrolase activity, such modulation may represent a potential mechanism underlying the observed metabolic improvements [43]. Further studies incorporating detailed bile acid profiling and gut microbial metabolomics are warranted to clarify this relationship.

The lack of significant changes in several systemic plasma markers, such as glucose, AST, and ALT (Table 4), in the HFD group after 8 weeks is consistent with the nature of our early-stage obesity model. Studies using long-term HFD feeding in C57BL/6 mice demonstrate that the onset of severe metabolic pathology, such as insulin resistance, liver triglyceride accumulation, and systemic inflammation, often occurs later, typically beyond 8 to 12 weeks of dietary intervention [44,45]. For instance, one study documented that significant insulin resistance and elevated hepatic triglycerides appeared after 12 weeks of a 60% HFD. Furthermore, several reports, consistent with our findings (Table 4), indicate that serum triglyceride levels and major circulating inflammatory cytokines often show little change or remain below detection limits in high-fat diet models, especially in C57BL/6 mice fed for 8 weeks [35,45].

Histological analysis of the epididymal fat revealed a significant reduction in adipocyte area per cell in the HFD + LP group compared with that in the HFD group. Furthermore, histogram analysis of the adipocyte size distribution revealed that HFD feeding resulted in a shift toward larger adipocytes, with a concomitant reduction in smaller adipocyte populations. Notably, LP06CC2 administration attenuated this shift, restoring the adipocyte size distribution pattern to a level comparable to that of the ND group. Previous studies have reported similar suppression of adipocyte hypertrophy by *Lactobacillus* administration [46,47], consistent with our results. These findings suggest that LP06CC2 not only reduces the adipocyte size but may also preserve or restore healthy adipose tissue morphology, which is closely associated with improved metabolic outcomes in obesity. In addition to adipose tissue, the liver plays a pivotal role in the pathophysiology of obesity. Histological evaluation of hepatic steatosis using NAS revealed clear pathological differences between the ND and HFD groups, albeit the changes were relatively mild. Unexpectedly, the livers of HFD-fed mice exhibited numerous small lipid droplets scattered throughout the hepatic parenchyma, indicating early-stage microvesicular steatosis [21]. Notably, supplementation of HFD-fed mice with LP appeared to attenuate the appearance of these lipid droplets. These findings suggest that LP may exert protective effects against the initial development of hepatic steatosis, potentially before increases in serum AST and ALT activity. We were unable to perform accurate automated quantification of lipid droplets because available segmentation tools misidentified non–lipid areas, including vascular lumina such as hepatic arteries, portal veins, and sinusoids. As a result, histological evaluation relied on NAS scoring by professional pathologists under blinded conditions. Further investigations are warranted to elucidate the mechanisms underlying these early hepatic changes and evaluate the long-term impact of LP on liver health in diet-induced obesity.

In the epididymal fat, characteristic changes in gene expression in response to HFD feeding were revealed, and these effects were partially or completely normalized by LP06CC2 supplementation. The expression levels of *Lpl* and *Pparγ*, key regulators of lipid uptake and adipogenesis, were significantly increased in the HFD-fed mice, but significantly suppressed in the HFD + LP group, suggesting that LP06CC2 alleviates adipocyte hypertrophy by modulating lipid accumulation pathways. Notably, the pro-inflammatory markers *Mcp-1*, *Il-6*, and *Scd1*, which are involved in lipid desaturation, were significantly upregulated in the HFD mice, and these increases were mitigated by LP06CC2 supplementation, indicating potential anti-inflammatory and lipid-normalizing effects. Likewise, *Ucp1* and *Ucp2*, which are involved in mitochondrial thermogenesis and energy expenditure, were significantly upregulated in the HFD mice and normalized in the LP06CC2-supplemented animals. However, the regulation of *Ucp* expression appears to be controversial, as previous reports have shown highly variable responses to HFDs depending on animal species, strain, tissue, and environmental conditions [48,49,50]. Interestingly, *Fas*, a critical enzyme in de novo lipogenesis, was significantly downregulated in the HFD-fed mice. This is also in contrast to many previous reports showing that Fas expression was elevated in diet-induced obesity models. The normalization trend observed in the LP06CC2-supplemented mice suggests that this probiotic strain may restore the lipogenic activity to homeostatic levels. Taken together, our results indicate that LP06CC2 supplementation modulates a range of adipocyte genes involved in lipid uptake, storage, mobilization, oxidation, and inflammation. Moreover, the unexpected expression patterns of *Fas*, *Ucp1/2*, *Hsl*, and *Cpt-1* highlight the complex and possibly time-dependent nature of adipose tissue remodeling in response to diet-induced obesity and probiotic intervention.

In our analysis of the alpha diversity of the intestinal microbiota, both the observed features and the Chao1 richness estimator [26] exhibited greater inter-individual variation in the HFD group than in the ND group, suggesting that HFDs induce heterogeneous changes in gut microbial richness. Although the Shannon diversity index [27] showed a slightly increasing trend in the HFD group compared with that in the ND group, this difference was not statistically significant. Notably, both the ND + LP and HFD + LP groups showed a significant increase in their Shannon indexes relative to that of the ND group. Although previous studies have reported that HFDs tend to disrupt the gut environment and decrease the alpha diversity [51,52,53], our current findings showed a modest increase in this indicator in the HFD group compared with that in the ND group. These results suggest that LP06CC2 supplementation may enhance both the richness and evenness of the gut microbiota, potentially alleviating HFD-induced dysbiosis. Although no significant differences in beta diversity were observed according to the weighted UniFrac distances [28], both the Bray–Curtis [29] and Jaccard distances [30] revealed significant group separation. This suggests that the microbial community underwent compositional changes in terms of relative abundance and the presence/absence of specific taxa, whereas the overall phylogenetic structure remained largely conserved. This pattern indicates that the probiotic intervention modulated the microbiota at the genus or species level without causing substantial shifts in dominant phylogenetic lineages.

The GSR-DB, a species-level taxonomy reference derived from the Genome Taxonomy Database, which provides higher species resolution and classification accuracy than conventional databases such as SILVA and Greengenes, was used in this study for 16S rRNA sequence classification [25]. *L. plantarum* is generally not a resident member of murine gut microbiotas, particularly under SPF conditions. Previous reports have indicated that LP is either absent or present at levels below the detection limit in untreated mice. In our study, LP was detected only in the ND + LP and HFD + LP groups, confirming that its administration via feed incorporation was successful. These findings indicate that our dietary supplementation protocol of 0.1% LP mixed into the feed effectively delivered the bacterial cells to the gut and allowed their transient colonization and detection. Among the microbial changes, those of *M. schaedleri* exhibited a particularly noteworthy pattern. Its abundance was significantly increased in the HFD group relative to that in the ND, ND + LP, and HFD + LP groups. LP06CC2 treatment suppressed this increase and restored the level close to that observed in the ND group. *M. schaedleri* is known to colonize the intestinal mucosa and is associated with inflammatory conditions, especially following HFD intakes [47]. Similar trends were observed for the Lachnospiraceae and Oscillospiraceae families and *A. mucosicola*; that is, these taxa were significantly enriched in the HFD group but suppressed in the HFD + LP group, with their abundance in the latter being comparable to that in the ND group. Lachnospiraceae and Oscillospiraceae are generally recognized as beneficial taxa producing SCFAs, particularly butyrate, in the gut. However, strain-level differences have been reported, with certain members exhibiting obesity-promoting or pro-inflammatory characteristics [51,54,55,56]. These findings suggest that the expansion of these taxa may play a role in early-stage obesity-associated gut dysbiosis and inflammation and that dietary supplementation with LP06CC2 may attenuate such microbial shifts. Further studies are warranted to explore the functional implications of these microbial changes in the pathogenesis of obesity.

Furthermore, Spearman’s correlation analysis provided insights into the relationship between the observed adipocyte profiles and specific gut taxa. We confirmed that the accumulation of epididymal fat mass and adipocyte size were positively correlated with the abundance of *M. schaedleri* and Lachnospiraceae. These specific taxa were previously shown to be enriched by HFD and suppressed by LP06CC2 treatment, suggesting that the HFD-induced microbial shifts are closely associated with adipose tissue pathology. The strong positive correlations observed among genes like *Pparγ* (a key regulator of adipogenesis) and inflammatory/thermogenic markers (*Ucp2*, *Il-6*, *Mcp-1*) reflect the complex metabolic state of the adipose tissue in this model. Specifically, the potent negative correlation between *Mcp-1* (pro-inflammatory marker) and *Fas* (lipogenic enzyme) may suggest that a high inflammatory status is inversely related to de novo lipogenesis in this early phase of obesity, potentially indicating metabolic stress consistent with previous studies that described it as being driven by inflammation and oxidative stress induced by nutrient overload [57,58]. Overall, the modulation of these specific bacterial populations by LP06CC2, which correlates highly with reduced fat accumulation, may represent a relevant mechanism through which the probiotic strain exerts its preventive effects.

This study has several limitations. First, only male mice were used, and potential sex-specific effects cannot be excluded. Second, the findings are based on a single *L. plantarum* strain, so strain-specific effects should be interpreted cautiously and not generalized to all *Lactobacillus* species. Third, although the gut microbiota composition and host metabolic parameters were analyzed, metabolomic data such as SCFAs and bile acids were not included, limiting our ability to directly link microbial changes with metabolic outcomes. Fourth, hepatic steatosis severity was assessed semi-quantitatively using the NAS on H&E sections, lacking the quantitative rigor of methods such as Oil Red O staining or automated imaging. Future studies incorporating both sexes, multiple strains, and integrated metabolomic profiling and quantitative hepatic histology will be essential to further elucidate the underlying mechanisms and translational potential in humans.

## 5. Conclusions

In conclusion, LP06CC2 displayed anti-obesity effects in a mouse model of mild diet-induced obesity generated via 8 weeks of HFD feeding. The LP06CC2 treatment suppressed adipocyte hypertrophy, changed the gut microbial diversity, and shifted the relative abundance of specific bacterial taxa. The confirmed positive correlations between the HFD-enriched taxa (e.g., *M. schaedleri* and Lachnospiraceae) and adipose tissue pathology strongly suggest that the LP06CC2-mediated microbial modulation is mechanistically linked to the attenuation of adipocyte hypertrophy, supporting its potential as a functional food component for early intervention in obesity. Future studies are warranted to explore the effects of LP06CC2 in moderate obesity models and elucidate its underlying mechanisms in greater detail. Moreover, its efficacy should be validated in human clinical trials.

## Figures and Tables

**Table 1:** Compositions of the various diets.

Component (g/kg)	ND	ND + LP	HFD	HFD + LP
Casein	200	200	187	187
Corn starch	529.5	528.5	212.5	211.5
Sucrose	100	100	100	100
Soybean oil	70	70	70	70
Lard	0	0	330	330
Cellulose	50	50	50	50
Choline bitartrate	2.5	2.5	2.5	2.5
L-Cystine	3	3	3	3
Mineral mix	35	35	35	35
Vitamin mix	10	10	10	10
*tert*-Butylhydroquinone	0.014	0.014	0.014	0.014
LP06CC2	0	1	0	1
Total	1000	1000	1000	1000

ND—normal diet; HFD—high-fat diet; LP—*Lactiplantibacillus plantarum* strain 06CC2 (LP06CC2).

**Table 2:** Physiological parameters.

	ND	ND + LP	HFD	HFD + LP
Final body weight (g)	27.80 ± 0.82	29.00 ± 0.87	30.20 ± 0.78	29.70 ± 0.91
Food intake (g/day)	4.57 ± 0.07 ^a^	4.62 ± 0.11 ^a^	2.53 ± 0.06 ^b^	2.64 ± 0.06 ^b^
Calorie intake (kcal/day)	18.40 ± 0.25 ^a^	18.20 ± 0.42 ^a^	14.20 ± 0.34 ^b^	14.80 ± 0.33 ^b^

Data are presented as the mean ± SEM for eight mice per group. ^a, b^ Values without any common letters are significantly different from each other (*p* < 0.05). ND—normal diet; HFD—high-fat diet; LP—*Lactiplantibacillus plantarum* strain 06CC2.

**Table 3:** Tissue masses.

(mg/g Body Weight)	ND	ND + LP	HFD	HFD + LP
Liver	30.40 ± 0.59	30.40 ± 0.98	27.50 ± 0.97	27.10 ± 1.01
Small intestine	20.70 ± 1.70	21.10 ± 1.61	20.70 ± 0.76	21.00 ± 0.97
Cecum	8.95 ± 1.75	7.26 ± 0.64	5.97 ± 0.62	6.17 ± 0.45
Large intestine	4.83 ± 0.69	3.80 ± 0.15	5.25 ± 1.08	4.10 ± 0.15
Epididymal fat	16.90 ± 2.37 ^ac^	13.80 ± 2.08 ^a^	29.70 ± 4.26 ^b^	25.60 ± 3.83 ^bc^
Perirenal fat	2.91 ± 0.59 ^ab^	1.79 ± 0.53 ^b^	3.88 ± 0.72 ^a^	3.24 ± 0.81 ^ab^

Data are presented as the mean ± SEM for eight mice. ^a, b^ Values without any common letters are significantly different from each other (*p* < 0.05). ND—normal diet; HFD—high-fat diet; LP—*Lactiplantibacillus plantarum* strain 06CC2.

**Table 4:** Plasma and hepatic lipid profiles of the various groups.

	ND	ND + LP	HFD	HFD + LP
Plasma				
Glucose (mg/dL)	132.60 ± 10.60	122.90 ± 9.40	122.90 ± 5.53	107.70 ± 6.50
TGs (mg/dL)	15.50 ± 1.89	14.30 ± 1.85	16.60 ± 2.13	13.30 ± 1.22
TC (mg/dL)	65.50 ± 4.20	72.30 ± 2.80	57.80 ± 3.08	63.60 ± 2.50
HDL-C (mg/dL)	47.10 ± 1.58	47.90 ± 1.08	47.10 ± 3.36	46.60 ± 3.12
LDL-C (mg/dL)	2.00 ± 0.00 ^ab^	1.63 ± 0.18 ^b^	2.50 ± 0.27 ^a^	1.88 ± 0.13 ^ab^
HDL-C/LDL-C	23.60 ± 0.79	30.80 ± 4.51	20.30 ± 2.35	26.30 ± 3.35
NEFA (µEq/dL)	790.30 ± 67.70^a^	824.10 ± 87.2 ^ab^	1013.60 ± 69.40 ^b^	994.40 ± 66.50 ^b^
AST (IU/L)	85.70 ± 12.40	92.90 ± 13.00	72.30 ± 3.40	82.30 ± 6.85
ALT (IU/L)	15.70 ± 1.21	16.90 ± 1.24	17.90 ± 0.58	18.40 ± 1.80
UA (mg/dL)	0.85 ± 0.09	0.90 ± 0.10	0.91 ± 0.12	0.84 ± 0.05
TBA (µmol/L)	1.29 ± 0.18	1.43 ± 0.30	14.50 ± 8.79	7.71 ± 6.38
FGF21 (pg/mL)	115.60 ± 33.20 ^a^	675.50 ± 268.40 ^b^	531.90 ± 111.20 ^b^	777.40 ± 331.30 ^ab^
Liver				
TGs (mg/g)	13.47 ± 1.37	12.10 ± 0.94	16.94 ± 2.91	12.88 ± 1.09
TC (mg/g)	6.33 ± 0.13	6.53 ± 0.14	6.59 ± 0.16	6.84 ± 0.13

Data are presented as the mean ± SEM for eight mice per group. Values without any common letters are significantly different from each other (*p* < 0.05). ND—normal diet; HFD—high-fat diet; LP—*Lactiplantibacillus plantarum* strain 06CC2; TGs—triglycerides; TC—total cholesterol; HDL-C—high-density lipoprotein-cholesterol; LDL-C—low-density lipoprotein-cholesterol; NEFA—non-esterified fatty acid; AST—aspartate aminotransferase; ALT—alanine aminotransferase; UA—uric acid; TBA—total bile acid; FGF21—fibroblast growth factor 21.

## Data Availability

The original contributions presented in the study are included in the article; further inquiries can be directed to the corresponding author.

## Supplementary Materials

The following supporting information can be downloaded at: https://www.mdpi.com/article/10.3390/nu17243855/s1, Figure S1: Changes in body weight; Table S1: List of primers for qPCR; Table S2: Detailed relative abundance of species-level fecal microbiota.
