# Supplementary material for: Lactiplantibacillus plantarum Strain 06CC2 Attenuates Fat Accumulation and Modulates the Gut Microbiota in a Mouse Model of Early-Stage Diet-Induced Obesity
Source: Nutrients. 2025 Dec 10;17(24):3855. doi: 10.3390/nu17243855 (PMC12736013; doi:10.3390/nu17243855)
Supplement: Supplementary file 1 [file nutrients-17-03855-s001.zip › nutrients-3960391-supplementary.pdf]

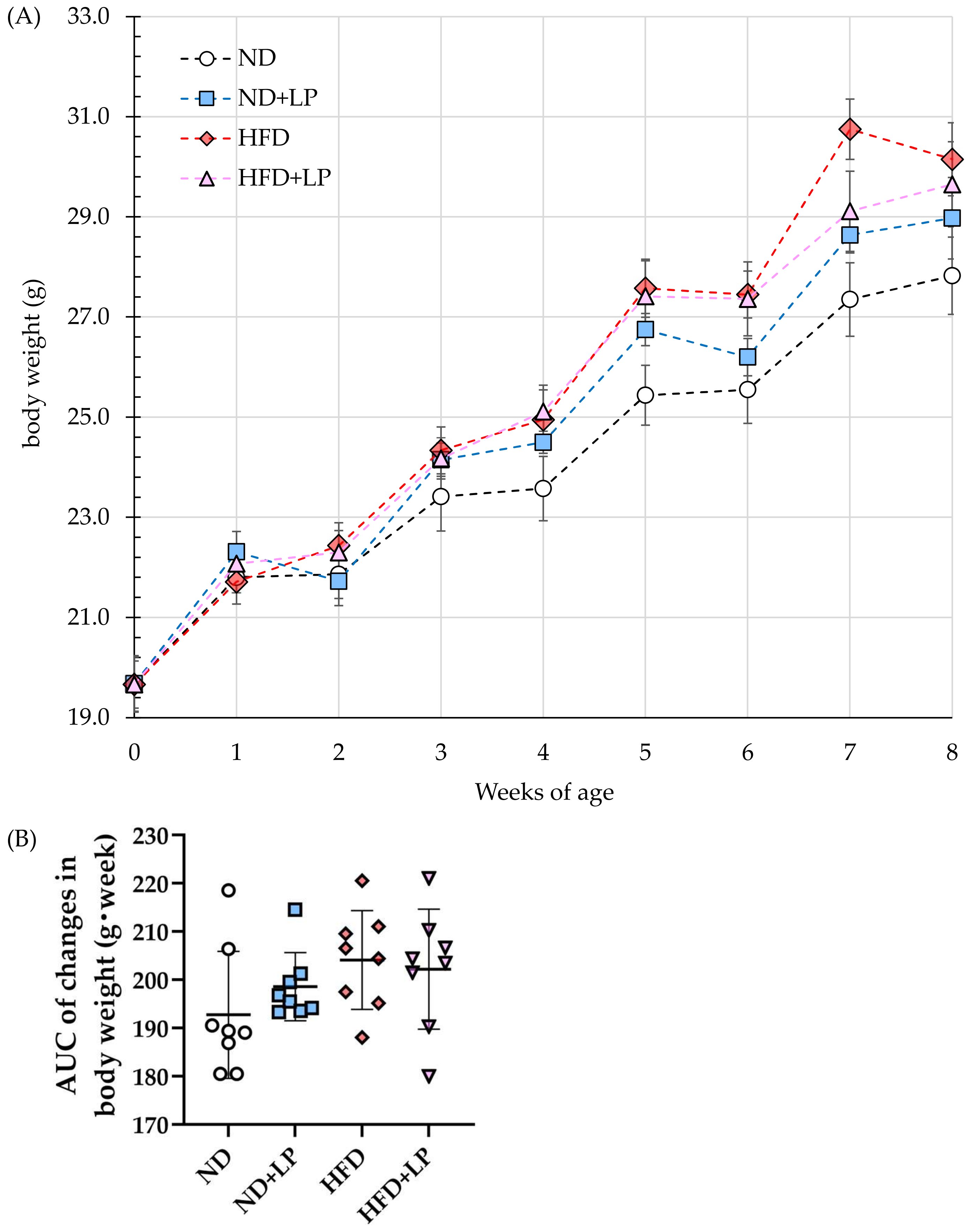

Figure S1. (A) Changes in body weight. (B) Area under the curve of changes in body weight. Data are presented as the mean ± SEM for 8 mice per group. ND, normal diet; HFD, high-fat diet; LP, *Lactiplantibacillus plantarum* strain 06CC2, AUC; area under the curve.

Table S1. List of primers for qPCR.

Gene	Forward primer (5' to 3')	Reverse primer (3' to 5')
Lpl	CATCGAGAGGATCCGAGTGAA	TGCTGAGTCCTTTCCCTTCTG
Pparγ	AGTGGAGACCGCCCAGG	GCAGCAGGTTGTCTTGGATGT
Mcp-1	CCACTCACCTGCTGCTACTCAT	TGGTGATCCTCTTGTAGCTCTCC
Il-6	CCACTTCACAAGTCGGAGGCTTA	CCAGTTTGGTAGCATCCATCATTTC
SCD1	TCAACTTCACCACGTTCTTCA	CTCCCGTCTCCAGTTCTCTT
Ucp1	AGGCTTCCAGTACCATTAGGT	CTGAGTGAGGCAAAGCTGATTT
Ucp2	CGAAGCCTACAAGACCATTGC	ACCAGCTCAGCACAGTTGACA
Fas	CTGGACTCGCTCATGGGTG	CATTTCCTGAAGTTTCCGCAG
Hsl	GGCTCACAGTTACCATCTCACC	GAGTACCTTGCTGTCCTGTCC
Cpt-1	TGAGTGGCGTCCTCTTTGG	TCAGCGAGTAGCGCATAGTCA
Il-1β	CGCAGCAGCACATCAACAAGAGC	TGTCCTCATCCTGGAAGGTCCACG
Tnf-α	TATGGCCCAGACCCTCACA	GGAGTAGACAAGGTACAACCCATC
Gapdh	TGTGTCCGTCGTGGATCTGA	TTGCTGTTGAAGTCGCAGGAG

Lpl, lipoprotein lipase; *Pparγ*, peroxisome proliferator-activated receptor gamma; *Mcp-1*, monocyte chemoattractant protein-1; *Il-6*, interleukin-6; *Scd1*, stearoyl-CoA desaturase; *Ucp1* and *Ucp2*, uncoupling proteins 1 and 2; *Fas*, fatty acid synthase; *Hsl*, hormone-sensitive lipase; *Cpt-1*, carnitine palmitoyltransferase 1; *Il-1β*, interleukin-1β; *Tnf-α*, tumor necrosis factor-alpha; *Gapdh*, glyceraldehyde-3-phosphate dehydrogenase.

Table S2. Detailed relative abundance of species-level fecal microbiota.

No.	Relative abundance (%)	ND	ND+LP	HFD	HFD+LP
		Mean ± SEM			
1	s_*Faecalibaculum_rodentium*	17.940 ± 2.577	14.273 ± 1.106	15.588 ± 2.201	12.525 ± 1.284
2	s_*Clostridium_celatum*	17.287 ± 1.814	12.479 ± 1.714	14.230 ± 1.583	14.846 ± 2.304
3	g_*Lactiplantibacillus_* sp.	0.000 ± 0.000	7.411 ± 0.335	0.000 ± 0.000	9.259 ± 0.842
4	s_*Phocaeicola_sartorii*	8.374 ± 1.747	11.572 ± 1.645	4.130 ± 0.491	3.760 ± 0.547
5	f_Lachnospiraceae	8.185 ± 0.852	10.271 ± 0.550	18.588 ± 1.260	14.622 ± 1.583
6	s_*Turicibacter_sanguinis*	6.832 ± 1.353	4.940 ± 0.999	2.868 ± 0.774	1.214 ± 0.233
7	s_*Ligilactobacillus_murinus*	3.587 ± 0.978	5.026 ± 1.742	2.769 ± 0.469	2.351 ± 0.184
8	f_Desulfovibrionaceae	2.499 ± 0.320	2.997 ± 0.199	3.282 ± 0.352	3.191 ± 0.097
9	s_*Prevotellamassilia_timonensis*	2.679 ± 0.419	3.049 ± 0.340	2.424 ± 0.332	2.517 ± 0.312
10	p_Bacteroidetes	2.657 ± 0.173	2.376 ± 0.275	2.016 ± 0.162	2.113 ± 0.073
11	s_*Akkermansia_muciniphila*	0.417 ± 0.339	0.030 ± 0.013	3.497 ± 1.138	4.029 ± 1.619
12	s_*Bacteroides_acidifaciens*	2.640 ± 0.227	3.541 ± 0.304	2.654 ± 0.340	2.558 ± 0.296
13	s_*Duncaniella_muris*	4.494 ± 0.575	3.849 ± 0.360	2.463 ± 0.119	2.534 ± 0.119
14	f_Muribaculaceae	1.143 ± 0.088	1.371 ± 0.105	1.005 ± 0.256	0.826 ± 0.162
15	s_*Mucispirillum_schaedleri*	0.687 ± 0.110	0.774 ± 0.119	1.577 ± 0.182	0.920 ± 0.170
16	s_*Acetatifactor_muris*	1.171 ± 0.265	1.759 ± 0.215	0.789 ± 0.346	0.967 ± 0.326
17	s_*Eubacterium_coprostanoligenes*	1.485 ± 0.181	1.786 ± 0.160	0.639 ± 0.124	0.633 ± 0.098
18	s_*Romboutsia_ilealis*	0.209 ± 0.071	0.106 ± 0.053	1.727 ± 0.382	2.908 ± 0.400
19	s_*Ruminococcus_gnavus*	1.756 ± 0.351	1.735 ± 0.090	3.820 ± 0.365	3.453 ± 0.401
20	s_*Lawsonibacter_asaccharolyticus*	0.637 ± 0.135	0.405 ± 0.041	1.907 ± 0.116	1.676 ± 0.225
21	o_Bacillales	1.338 ± 1.058	0.105 ± 0.072	1.135 ± 0.432	0.068 ± 0.059
22	s_*Flintibacter_butyricus*	0.783 ± 0.095	0.943 ± 0.092	1.539 ± 0.121	1.674 ± 0.175
23	s_*Parasutterella_excrementihominis*	0.722 ± 0.189	0.419 ± 0.139	0.322 ± 0.099	0.261 ± 0.052
24	o_Eubacteriales	1.593 ± 0.134	1.614 ± 0.138	2.287 ± 0.462	2.230 ± 0.227
25	s_*Paramuribaculum_intestinale*	2.149 ± 0.176	2.354 ± 0.187	1.543 ± 0.212	1.060 ± 0.261
26	g_*Limosilactobacillus*	1.290 ± 1.009	0.006 ± 0.004	0.035 ± 0.024	0.162 ± 0.084
27	f_Oscillospiraceae	0.965 ± 0.085	1.040 ± 0.082	1.612 ± 0.205	1.197 ± 0.120
28	s_*Adlercreutzia_caecimuris*	0.247 ± 0.052	0.214 ± 0.030	0.849 ± 0.072	0.671 ± 0.058
29	f_Atopobiaceae	0.000 ± 0.000	0.000 ± 0.000	0.000 ± 0.000	1.169 ± 0.488
30	s_*Anaerotignum_lactatifermentans*	0.204 ± 0.032	0.232 ± 0.028	0.288 ± 0.020	0.272 ± 0.026
31	others	6.030 ± 2.025	3.323 ± 0.206	4.416 ± 0.369	4.333 ± 0.372
